# REVOLUTIONIZING COGNITIVE ASSESSMENT: THE POWER OF DIGITAL BIOMARKERS AND ECOLOGICALLY VALID METHODS

**DOI:** 10.1093/geroni/igad104.1260

**Published:** 2023-12-21

**Authors:** Nelson Roque, Martin Sliwinski, Mindy Katz, Cay Anderson-Hanley

**Affiliations:** Pennsylvania State University, University Park, Pennsylvania, United States; Pennsylvania State University, State College, Pennsylvania, United States; Albert Einstein College of Medicine, Bronx, New York, United States; Union College, Schenectady, New York, United States

## Abstract

Digital technologies have allowed neuropsychologists to utilize process-based approaches to assess cognitive function and change. Traditional methods of neuropsychological assessment involve obtaining single scores (often composites) to represent cognitive function. However, modern process-based techniques involve the use of digital data collection to acquire insights into both the manner and reasoning behind an individual’s performance. The present study will share insights into the task development and validation of active and passive digital cognitive assessments from two longitudinal studies of aging: (1) a study of non-dementia participants (N=322), ultra-brief mobile cognitive tests were administered up to six times per day for two weeks; and (2) a neuro-exergaming study (N=31) enrolling patients with MCI and their co-residing study partner, where participants used a study-provided tablet to complete a neuro-exergaming protocol and digital cognitive assessments (e.g., digital trailmaking and eStroop). We analyzed both traditional and process-based metrics in two studies, including mean response time and patterns of inaccuracy. Study one found significant correlations between MCI status and metrics for survey completion time, working memory, and processing speed. Study two found weak to moderate correlations between cognitive function measures from digital and paper-based neuropsychology. Our findings suggest that remote self-administered digital measures can detect subtle cognitive changes. The event-level data, which is collected through a process-based approach, has enormous untapped potential for discovering and confirming novel digital biomarkers of cognitive health and progression that can be monitored beyond clinical settings.

